# Seek and destroy: targeted adeno-associated viruses for gene delivery to hepatocellular carcinoma

**DOI:** 10.1080/10717544.2016.1247926

**Published:** 2017-02-06

**Authors:** Bijay Dhungel, Aparna Jayachandran, Christopher J. Layton, Jason C. Steel

**Affiliations:** 1 Gallipoli Medical Research Institute, Greenslopes Private Hospital, Brisbane, QLD, Australia,; 2 School of Medicine, The University of Queensland, Brisbane, QLD, Australia,; 3 University of Queensland Diamantina Institute, Translational Research Institute, Woolloongabba, QLD, Australia, and; 4 Ophthalmology Department, Gallipoli Medical Research Institute, Greenslopes Private Hospital, Brisbane, QLD, Australia

**Keywords:** AAV, HCC, targeted gene delivery, gene therapy transcriptional targeting, transductional targeting, capsid modification, tumor specific promoters

## Abstract

Hepatocellular carcinoma (HCC) is the most common form of primary liver cancer with high incidence globally. Increasing mortality and morbidity rates combined with limited treatment options available for advanced HCC press for novel and effective treatment modalities. Gene therapy represents one of the most promising therapeutic options. With the recent approval of herpes simplex virus for advanced melanoma, the field of gene therapy has received a major boost. Adeno-associated virus (AAV) is among the most widely used and effective viral vectors today with safety and efficacy demonstrated in a number of human clinical trials. This review identifies the obstacles for effective AAV based gene delivery to HCC which primarily include host immune responses and off-target effects. These drawbacks could be more pronounced for HCC because of the underlying liver dysfunction in most of the patients. We discuss approaches that could be adopted to tackle these shortcomings and manufacture HCC-targeted vectors. The combination of transductional targeting by modifying the vector capsid and transcriptional targeting using HCC-specific promoters has the potential to produce vectors which can specifically seek HCC and deliver therapeutic gene without significant side effects. Finally, the identification of novel HCC-specific ligands and promoters should facilitate and expedite this process.

## Introduction

Liver cancer is the fifth most common cancer and causes one third of cancer related deaths worldwide (Siegel et al., 2015). With an estimated 746 000 deaths in 2012 alone; its incidence is rising globally (Siegel et al., 2015; Zhang et al., 2015). Difficulty in the effective management of liver cancer is a result of a combination of poor diagnosis and prognosis and limited therapeutic options.

Hepatocellular carcinoma (HCC), which accounts for over 80% of liver cancer, mostly develops in the cirrhotic liver (El-Serag & Rudolph, 2007). Liver cirrhosis may be caused by a number of factors including chronic viral hepatitis, alcohol abuse, inherited metabolic maladies (e.g. hemochromatosis) and nonalcoholic fatty liver disease (Liver & Cancer, 2012). The wide range of predisposing conditions coupled with the overlap of hepatic clinical symptoms generally makes early diagnosis of HCC difficult (Forner et al., 2012; Liver & Cancer, 2012). As a result, most patients are diagnosed at an advanced stage and have a poor prognosis (Forner et al., 2012). Even with improved surveillance and detection, overall survival rate for HCC has not improved, highlighting the poor status of current therapy (Liver & Cancer, 2012; Siegel et al., 2015).

Liver transplant is the best option to cure both HCC and cirrhosis but its suitability is limited by tumor stage and patient’s health status as well as the lack of donors, difficulty to obtain histological parameters before transplant, problems with graft rejection and the possibilities of opportunistic infections due to immunosuppression (Liver & Cancer, 2012; Waghray et al., 2015). Tumor resection or ablation is an option available for early stage HCC but recurrence or the development of new tumors in the diseased liver is frequently observed (Waghray et al., 2015). Additionally, there is a significant risk of mortality and morbidity when using these methods because of the underlying condition of the cirrhotic liver (Forner et al., 2012). Sorafenib, a multikinase inhibitor, is the only drug available for systemic therapy of HCC (Llovet et al., 2008; Waghray et al., 2015); however its efficacy is limited with an overall increase in survival of 2–3 months (Cheng et al., 2009; Flaherty & Sun, 2009). In addition, it can only be used in patients with preserved liver function (Torrecilla & Llovet, 2015). With the incidence of liver cancer growing and a lack of an effective treatment options, there is a pressing need for the development of novel targeted therapies against HCC. Gene therapy, which involves the transfer of a therapeutic gene to diseased cells or tissues using a vector, is one attractive treatment strategy (Amer, 2014).

## AAV gene therapy for HCC

**Figure 1.**
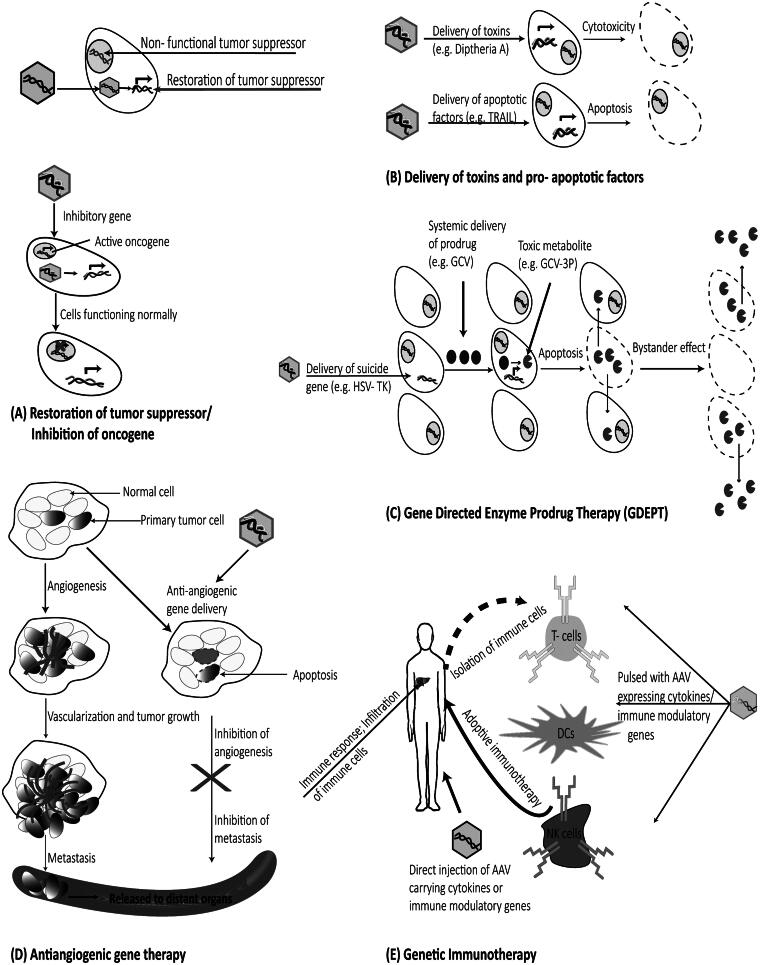
Strategies used for gene therapy of HCC. (A) Restoration of tumor suppressor genes or inhibition of oncogenes may restore normal functioning of the tumor cells. (B) Direct administration of recombinant rAAV expressing toxins or apoptotic factors like TRAIL can lead to tumor cytotoxicity and/or apoptosis. (C) GDEPT is a two-step process to induce tumor cell death. Tumor cells are first transduced with rAAV expressing suicide gene followed by systemic administration of prodrug which is metabolized by the transduced cell into a toxic metabolite. (D) Anti-angiogenic gene therapy using AAV vectors can inhibit formation of new blood vessels, ultimately leading to tumor apoptosis and inhibition of metastasis. (E) Delivery of cytokines and immunomodulatory genes either using AAV vector or immune cells transduced with rAAV vectors harboring cytokines (adoptive immunotherapy) to tumor cells triggers an anti-tumor immune response via recruitment of immune cells.

An ideal gene therapy vector should exhibit properties of low pathogenicity and an ability to maintain sustained levels of therapeutic gene expression in a wide variety of target tissue/cell and induce minimal activation of the immune system. Adeno-associated virus based vectors (AAVs) have many of these characteristics (Luo et al., 2015). For HCC gene therapy, a number of preclinical studies have been conducted with AAV vectors to establish their effectiveness (Table 1). These strategies include restoration of tumor suppressors, delivery of cytotoxic genes, gene directed enzyme prodrug therapy (GDEPT), inhibiting angiogenesis, and genetic immunotherapy. The principles of each of these strategies have been illustrated in Figure 1. There are, however, a few limitations of AAV vectors which need to be addressed for effective HCC gene therapy.

**Table 1.** Gene therapy studies for HCC using AAV based vectors.

Therapeutic gene/intervention	Strategy/combination	Target	Results/comments	Ref.
HSV-TK	Suicide gene therapy	HCC cell lines	Selective killing of HCC cells using liver-specific promoter and tumor specific enhancer	(Su et al., 1996)
HSV-TK	Suicide gene therapy	Mouse model	Reduction of tumor growth and observation of by stander effect	(Su et al., 1997)
HSV-TK	Suicide gene therapy	HCC cell lines/mouse model	Use of a liver specific promoter combined with posttranscriptional regulation to achieve HCC-specific HSV-tk expression; reduction in tumor growth and low toxicity	(Della Peruta et al., 2015)
p53	Combination of rAAV-p53 with chemotherapy	HCC cell lines	Increased permissiveness of HCC cells following doxorubicin treatment; synergistic cytotoxic effects were seen	(Chen et al., 2008)
p53/HGFK1	Combination of rAAV-HGFK1 with rAdV-p53	HCC cell lines, mouse and rat models	Decreased proliferation of HCC cells *in vitro*; prolonged survival in animal models by induction of tumor cell death and antiangiogenesis	(Shen et al., 2008a)
HGFK1	Anti-angiogenesis rAAV-HGFK1	HCC cell lines and rat HCC model	Increased survival, tumor cell death by induction of apoptosis and antiangiogenesis, prevention of metastasis	(Shen et al., 2008b)
Angiostatin	Anti-angiogenesis rAAV-angiostatin	Mouse model	Stable, high-level expression of angiostatin for at least six months; inhibition of metastasis and extensive tumor apoptosis; increased survival	(Xu et al., 2003)
Endostatin	Anti-angiogenesis (rAAV-endostatin) combined with chemotherapy	HCC cell lines, mouse model	Increased transduction and high level of endostatin expression when combined with etoposide as compared to control or monotherapy groups both *in vitro* and *in vivo*; antitumor and antiangiogenic responses were observed along with increased survival in animal model	(Hong et al., 2004)
Kallistatin	Anti-angiogenesis	HCC cell lines, mouse models	Suppression of tumor cell proliferation and apoptosis via inhibition of angiogenesis	(Tse et al., 2008)
Kringle domains of apo(a)	Anti-angiogenesis	HCC cell lines, mouse model	Inhibition of angiogenesis, induction of tumor apoptosis and prolonged survival in animal model	(Lee et al., 2006)
TRAIL	Induction of tumor apoptosis	Mouse model	Prolonged survival; induction of tumor apoptosis and prevention of metastasis	(Ma et al., 2005a)
TRAIL/insulin	Induction of apoptosis	Mouse model	Oral administration of rAAV-TRAIL fused to secretion signal peptide of insulin demonstrated reduced tumor growth and non-toxicity to normal hepatocytes	(Ma et al., 2005b)
TRAIL	Induction of apoptosis	HCC cell lines, mouse model	TRAIL under the control of hTERT promoter (rAAV-hTERT-TRAIL) lead to cancer specific TRAIL expression and tumor apoptosis	(Wang et al., 2008)
TRAIL	Induction of apoptosis combined with chemotherapy	HCC cell lines, mouse model	Systemic administration of rAAV-hTERT-TRAIL lead to tumor-specific TRAIL expression; combinatorial therapy with 5-FU lead to enhanced tumor cell death	(Zhang et al., 2008)
TRAIL	Induction of apoptosis combined with chemotherapy	HCC cell lines, mouse model	Enhanced TRAIL expression was observed in tumor cells following treatment with cisplatin. Increased tumor cell death and apoptosis was observed *in vivo*	(Wang et al., 2010)
miR26a	Induction of tumor apoptosis	HCC cell lines, mouse model	Systemic administration of rAAV-miR26a lead to tumor specific induction of apoptosis, inhibition of tumor proliferation and no toxicity	(Kota et al., 2009)
Apoptotin/IL24	Induction of apoptosis combined with cytokine therapy	HepG2, mouse model	Synergistic antitumor effects of apoptotin and IL24 was observed by induction of apoptosis	(Yuan et al., 2013)
Secondary lymphoid tissue chemokine (SLC)	Genetic immune therapy	Hepa1-6, mouse model	Hepa1-6 pretreated with rAAV-SLC was injected in mouse model; delayed tumor progression and strong anti-tumor immune response by infiltration of DCs and activated T-cells were observed	(Liang et al., 2007)
IL15	Genetic immune therapy	Mouse model	Prolonged survival; significant antitumor response mainly dependent on NK cells; no observable liver toxicity	(Chang et al., 2010)
IL12	Genetic immune therapy	Mouse model	Increased survival, inhibition of metastasis, decrease in tumor vessel density and antitumor response by infiltration of NK cells, activated T cells and NKT cells. No such effects were observed with IL23 and IL27	(Lo et al., 2010)
IL12/IFN gamma	Genetic immune therapy	Mouse model	Liver specific, tet-on inducible AAV system with liver-specific promoter was used to express IL12 *in vivo*. Prolonged survival, significant antitumor response and memory T cell response was observed	(Vanrell et al., 2011)
AFP	*Ex vivo* gene therapy	HepG2 and BEL7402	DCs generated from mononuclear cells transduced with rAAV-AFP and cultured along with GM-CSF and IL4 displayed enhanced ability to activate cytotoxic T-cells	(Du & Yu, 2011)
LacZ (reporter)	Evaluation of the effects of chemo and radiotherapy on rAAV tumor transduction	HCC cell lines, mouse and rat models	Enhanced transduction was observed with radio and chemotherapy *in vitro*, however, only radiation was able to show similar effects in animal models	(Peng et al., 2000)

The host immune responses are possibly the biggest obstacle for successful gene therapy; the extent of which depends on the target organ, the route of vector administration, the transgene and the vector itself (Asokan et al., 2012). Observations from clinical trials have revealed that immune responses against AAV are comparatively low in immune-privileged organs like the eye and the brain, however, they can be more problematic in other organs such as the liver, muscle or lung (Ferreira et al., 2014).

Neutralizing antibodies (NAbs) to the AAV capsid may be a limiting factor to successful gene therapy. The NAbs resulting from natural exposure to wild-type AAV cross-react with AAV vectors after systemic administration. Calcedo et al. have reported that natural exposure to AAV leads to the development of NAbs very early in life (∼2 years) (Calcedo et al., 2011). Similar studies to assess the level of preexisting antibodies against AAV vectors have demonstrated that the frequency of antibodies against AAV could be as high as 70% in healthy individuals (Calcedo et al., 2009; Boutin et al., 2010). Even though the most frequent of these antibodies are directed against AAV serotype 2, cross-reactivity between wide ranges of serotypes due to conserved amino acid sequences in the viral capsid has been reported (Calcedo & Wilson, 2013; Gurda et al., 2013). These antibodies not only decrease the efficacy of gene therapy by neutralization but also limit the possibility of vector re-administration.

In addition to the NAbs, capsid specific CD8^+^ T-cell responses have been observed against AAV in clinical trials (Mingozzi & High, 2011,2013; George & Fogarty, 2016). Results from clinical gene therapy for hemophilia with AAV expressing factor IX (FIX) indicate that the development of capsid specific T-cell response is dependent on the dose of the vector (Manno et al., 2006; High & Aubourg, 2011; Mingozzi & High, 2011,2013; George & Fogarty, 2016). Similar capsid-specific T-cells were observed in a clinical trial against lipoprotein lipase deficiency (LPLD) where nine out of 14 subjects who received a mild dose of T-VEC (1 × 10^11^ vgs/kg) developed T-cell responses to the vector (Ferreira et al., 2014). This activation of cellular immunity leads to the destruction of transduced cells and ultimately to a loss of expression of therapeutic gene. This observation was different from preclinical studies in animal models including mice and non-human primates (Manno et al., 2006; Mingozzi & High, 2013) where T-cell mediated cellular immune response was not observed. Notably, a canine model of hemophilia showed stable and long-term expression of FIX for over nine years (Niemeyer et al., 2009; Mingozzi & High, 2011). In contrast, clinical trials have reported a correlation between the levels of capsid specific T-cells in peripheral blood and a decrease in the expression of FIX. Interestingly, only a minimal immune response against the therapeutic gene was observed in these trials (Ferreira et al., 2014; Arruda & Samelson-Jones, 2015; George & Fogarty, 2016).

To address the issue of host immune responses against AAV, a number of potential strategies are under development and have been extensively reviewed elsewhere (Masat et al., 2013; Mingozzi & High, 2013; Arruda & Samelson-Jones, 2015; Tse et al., 2015; George & Fogarty, 2016). Exclusion of patients with high levels of preexisting antibodies against AAV, increasing the vector dose to overcome NAbs, immunosuppression, plasmapheresis and the use of vector decoys are some of the strategies that are currently under investigation (Mingozzi & High, 2013; Tse et al., 2015). However, none of these strategies provide a long-term solution. The widespread nature of NAbs against AAV in humans and the poor sensitive methods for the detection of antibodies currently make the exclusion strategy impractical (Calcedo et al., 2009; Boutin et al., 2010; Mingozzi & High, 2013). Increasing the dose of vectors and use of decoys may lead to an increase in antigen load and subsequently, the induction of capsid specific T-cell responses (Mingozzi & High, 2013; Arruda & Samelson-Jones, 2015). Immunosuppression and plasmapheresis are impractical as a long term solutions as they require frequent intervention which is not feasible in HCC patients with poor liver function (Schlitt et al., 2011; Tse et al., 2015).

The use of AAV vectors which are specific to HCC at both transductional and transcriptional level might be able to overcome many of these obstacles and produce better therapeutic outcomes. First, capsid modification of the vector to increase HCC tropism may lead to a requirement of lower vector dose addressing the issue of cellular immune response against the capsid. Moreover, any capsid specific T-cell response would lead to the clearance of transduced tumor cells. Additionally, identification and mutation of sites in the capsid recognized by NAbs could solve the problem of preexisting immunity and re-administration. Mutations in the capsid of AAV5 have been shown to increase the resistance to NAbs directed against the wildtype virus (Afione et al., 2015). Secondly, the use of tumor specific promoters (TSPs) to limit the expression of therapeutic gene in HCC can minimize off-target effects. HCC specific promoters like alpha fetoprotein (AFP) have been used to express therapeutic genes in HCC-specific manner (Su et al., 1996; Ma et al., 2010). Steel et al. have shown that orally administered AAV5 is distributed predominantly in the liver (Steel et al., 2013). The combination of these AAV serotypes displaying a strong liver tropism like AAV8 (Zincarelli et al., 2008) with HCC-specific promoters could lead to an enhanced therapeutic gene expression in a HCC-specific manner.

## Modification of AAV vectors

### Transductional targeting of AAV

With the increase in understanding of vector biology and vector–host interactions, delineation of viral life cycle has led to the elucidation of steps important for viral tropism. Viral binding to cell surface receptor by capsid–receptor interaction is the first step in this process. Identification of sites on the viral capsid which are responsible for binding cellular receptors has made it possible to explore approaches that could ablate the natural broad tropism of the virus and redirect vectors to target cells (Afione et al., 2015; Büning et al., 2015; Liu et al., 2015). Furthermore, the identification of sites in the AAV capsid that can tolerate mutations, insertions and deletions allow for guided genetic capsid manipulation for cell-type specific redirection. The principles and efficacy of modifying viral capsids either by manipulating these sites or by inserting ligands specific to target-cell receptors have been reported for wide variety of cell targets (Kwon & Schaffer, 2008; Sallach et al., 2014; Büning et al., 2015; Liu et al., 2015).

Cellular transduction with AAV vectors starts with the binding of the vector at the cell surface (Bartlett et al., 2000) which is largely regulated by the capsid of the virus (Vandenberghe et al., 2009). The cell and tissue tropism of a particular serotype is dependent on its capsid as evident from the comparison of hepatic transduction efficiencies of six different serotypes (Grimm et al., 2006). The broad tropism inherent with AAV vectors is a disadvantage for gene therapy where targeted transgene expression is required. The modification of the vector capsid is, thus, an important area of research for targeted gene therapy.

Different serotypes of AAV exploit varieties of cell surface glycans for attachment to the cell surface, and with about 100 different known serotypes of AAV (Kwon & Schaffer, 2008), complete delineation of tropism and cell surface receptor for each of these serotypes remains an exciting field of investigation. AAV2, the most widely used AAV serotype for gene therapy, primarily uses cell surface heparan sulfate proteoglycan (HSPG) which is expressed in different cell types (Summerford & Samulski, 1998). AAV2 has been shown to efficiently transduce varieties of cells and tissues including hepatocytes, airway epithelium, cardiac muscle, skeletal muscle and brain tissue (Michelfelder & Trepel, 2009). In preclinical studies, liver tropism has been reported for AAV serotypes 5 (Davidoff et al., 2005; Steel et al., 2013), 6 (Jiang et al., 2006), 8 (Davidoff et al., 2005; Gao et al., 2006) and 9 (Sarkar et al., 2006). Interestingly, AAV serotype 3, which has been shown to have weak tropism for liver (Zincarelli et al., 2008; Cheng et al., 2012), exhibited strong tropism for HCC cells (Cheng et al., 2012) due in part to hepatocyte growth factor receptor (HGFR), a co-receptor for AAV3 (Ling et al., 2010), being overexpressed on HCC (Luo et al., 1999; Cheng et al., 2012). Exploiting this natural tropism of certain serotypes for HCC-specific transgene expression is a potential strategy of targeted gene therapy as shown by the efficacy of suicide gene therapy of disseminated HCC by AAV8 harboring a liver-specific promoter in combination with a HCC-specific miR122a binding sequence (Della Peruta et al., 2015).

Pseudotyping the genome of one AAV serotype into the capsid of another is another strategy that has been employed to achieve cell specific gene expression. A study conducted by Grimm et al. demonstrated that the capsid and not the genome is responsible for liver tropism, and provides the rationale for pseudotyping (Grimm et al., 2006). An enhanced expression of transgene using pseudotyped vectors have been reported for different cells and organs including skeletal muscle (Chao et al., 2000), dermal fibroblasts (Balaji et al., 2013), neurons (Alisky et al., 2000), eye (Auricchio, 2003), heart (Pacak et al., 2006) and more importantly in the liver (Grimm et al., 2003). The genome of AAV2 is the most widely studied and engineered of all the serotypes. It has been used for cell-specific therapeutic gene expression using regulatory elements like promoters and enhancers (Weitzman & Linden, 2011; Balakrishnan & Jayandharan, 2014). AAV2 genome containing therapeutic gene under the control of HCC-specific regulatory elements could potentially be pseudotyped with serotypes like AAV3 which have greater HCC-tropism. Another potential strategy is the generation of mosaic or chimeric capsid by mixing capsid subunits from different serotypes. AAV vectors with chimeric capsids have been reported to have enhanced transduction in endothelial cells (Stachler & Bartlett, 2006), muscle and liver (Hauck et al., 2003) when compared to parental serotypes.

These strategies, however, fail to address the obstacles posed by the host immune response (Waehler et al., 2007; Michelfelder & Trepel, 2009) and even if an enhanced HCC-cell transduction is achieved, the issue with general broad tropism of any new serotype would still remain a concern. In addition, the use of pseudotyped and chimeric vectors is limited by the availability of AAV serotypes with natural HCC-tropism. Targeting AAV using HCC-specific ligands is one strategy that might address some of these unresolved problems. The discovery of ligands which specifically bind to HCC (Du et al., 2006; Lo et al., 2008) will facilitate this process. This ligand-receptor mediated cell targeting can be achieved either by genetic modification of the capsid or by non-genetic means (Waehler et al., 2007; Sen et al., 2013; Sen, 2014).

Non-genetic means of capsid targeting can be achieved with adapter proteins such as bispecific antibodies which can bind to both viral capsid and the target cell (Waehler et al., 2007). To transduce megakaryocyte cells which are non-permissive to AAV, a bispecific antibody interacting with both the viral capsid and cell-specific receptor has been tested, and demonstrated a reduction of natural tropism of the virus and increase in selectivity of 70-fold (Bartlett et al., 1999). An alternate strategy is the genetic modification of the capsid that facilitates non-genetic targeting of the vector, for example, the insertion of the immunoglobulin binding domain of protein A into the capsid which binds of the Fc region of an antibody while allowing the antibody to retain its targeting motif. This method of targeting has been used in AAV to improve transduction of hematopoietic cell lines, where transduction was dependent on the binding of the inserted domain with cell surface antibodies (Ried et al., 2002). Similarly, biophysical probes and targeting ligands have been conjugated in a site-specific manner to biotin accepter peptide (BAP)-modified AAV using the biotin ligase enzyme. The transduction efficiency of the BAP-modified vector with integrin targeting peptide was found to be significantly higher in endothelial cells compared to the wild-type and an enhanced ability to deliver reporter gene to the tumor vasculature was observed in a mouse model of ovarian cancer (Stachler et al., 2008). Non-genetic targeting of AAV capsid in this way for HCC itself has not yet been reported. These methods do have limitations of technical complexity, destabilization of the vector complex, and difficulty of high-scale vector production (Büning et al., 2015; Liu et al., 2015).

**Figure 2.**
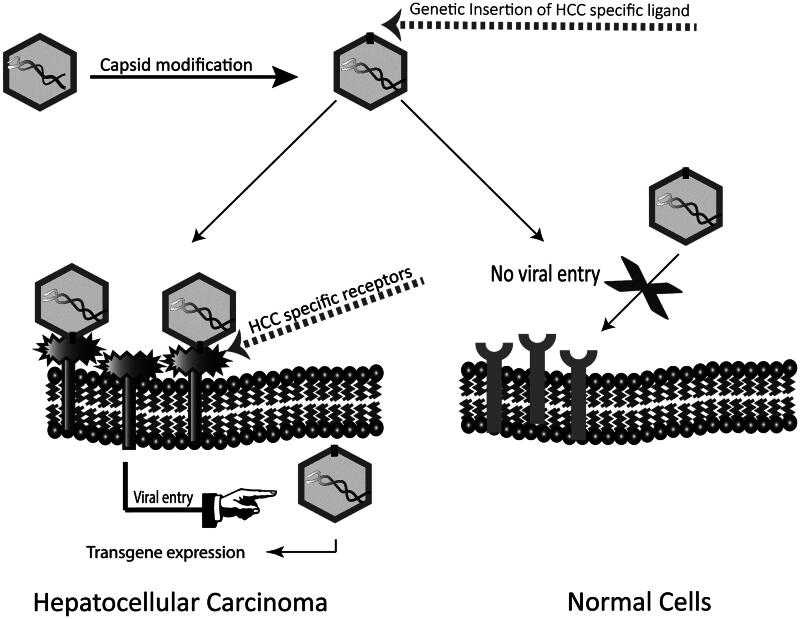
Capsid modification of AAV by insertion of HCC-binding ligands. Insertion of HCC-binding peptides at sites of AAV capsid which tolerate insertions without affecting viral life cycle can change the natural tropism of the vector and retarget it to HCC.

With the knowledge of molecular structure of the virus capsid (Xie et al., 2002) and identification of sites responsible for interaction with the receptor (Kern et al., 2003; Opie et al., 2003; Afione et al., 2015), genetic insertion of HCC-specific ligands at sites which tolerate manipulation is possible. Figure 2 depicts the principles of this possible method of manufacturing HCC-targeted AAV vectors.

The first study identifying potential sites in AAV2 which could facilitate peptide insertion without disrupting viral life cycle was conducted by Girod et al. Insertion of a L14 peptide which binds to the laminin receptor at amino acid 587 was found to change the natural tropism of the vector in an L14 dependent manner (Girod et al., 1999). Melanoma cell line B16F1025, which is non-permissive to wild-type AAV virus, was efficiently transduced with the mutant (Girod et al., 1999). Similarly, an enhanced and ligand specific endothelia cell transduction was observed when endothelial-specific ligand was inserted at the same site (Nicklin et al., 2001). For Kaposi sarcoma and rhabdomyosarcoma cell lines which express high levels of CD13, a binding ligand was inserted at amino acid position 587 to achieve cell-specific transduction (Grifman et al., 2001). In another study, Wu et al. identified additional sites in the AAV2 capsid which tolerated insertion. Insertion of peptide specific to serpin receptor at positions 34 and 138 led to 62 and 15-fold increase in transduction for IB3 cells respectively (Wu et al., 2000). Similarly, an increase in the transduction of breast cancer tissues was observed after insertion of peptide ESGLSQS at positions 590 and 589 of AAV8 and AAV9, respectively (Michelfelder et al., 2011). As an alternative to genetic insertion of cell-specific peptides, the development of AAV libraries based on capsid DNA shuffling, error prone PCR and directed evolution of known AAV capsid serotypes are being developed (Kotterman & Schaffer, 2014). The incorporation of designed ankyrin repeat protein (DARPins) libraries into the capsid has also been examined to target AAV. An ankyrin repeat protein 9.29 which binds with high affinity to human epidermal growth factor receptor/neu (HER2/neu), a receptor tyrosine kinase overexpressed in tumor cells has been fused with AAV capsid to ablate the natural tropism and re-direct the vector to HER2 positive tumor cells (Münch et al., 2013). The principles and relative merits and demerits of these strategies have been reviewed in a number of publications (Vandenberghe et al., 2009; Kotterman & Schaffer, 2014; Büning et al., 2015; Liu et al., 2015). These methods can not only increase the cell-specific transduction with AAV but can also generate vectors which can evade host immune responses (Maheshri et al., 2006; Li et al., 2016).

Masheri et al. constructed mutant vectors using capsid mutant library generated by error prone PCR. These vectors were subsequently incubated with pre-immunized rabbit sera and found to have an enhanced resistance to preexisting NAbs compared to the wild-type vector *in vivo* (Maheshri et al., 2006). More recently, a novel approach to generate patient-specific NAb escaping AAV mutants has been reported for muscle gene delivery (Li et al., 2016). In this study, NAb escaping AAVs were isolated from patients who had received AAV harboring mini-dystrophin gene during the Phase I clinical trial of Duchenne muscular dystrophy (DMD). The *in vivo* selection of these mutants resulted in isolation of AAVs that displayed high muscle tropism and superior ability to evade host immune responses (Li et al., 2016). For manufacturing AAV vectors with high tropism for liver, Lisowski et al. created and screened AAV capsid mutant library from 10 different serotypes (Lisowski et al., 2014). One of the chimeras composed of five different parental capsids displayed improved hepatocyte transduction efficiency. In addition, this mutant was able to transduce HCC cells in culture and in a xenograft model at higher efficiencies compared to any wild type serotype (Lisowski et al., 2014). These studies have demonstrated the possibility of integrating capsid modification techniques for the manufacture of clinically relevant AAV vectors to deliver therapeutic genes against HCC.

### Transcriptional targeting of AAV

The ablation of natural tropism and retargeting AAV to HCC by capsid modification has the potential to overcome many of the obstacles for successful gene therapy, however, a complete ablation of viral tropism is highly unlikely in practice, and therefore of equal importance is the transcriptional targeting of AAV to maintain target-cell specific therapeutic gene expression to limit off-target effects and toxicity to normal cells. A tightly regulated HCC-specific expression of therapeutic gene can minimize any off-target effects and toxicity of normal cells. With the ease of manipulation of AAV genome, transcription targeting of AAV with HCC-specific promoters can be used for this purpose.

During the process of malignant transformation there are significant changes in the expression of many genes induced by genetic and epigenetic factors. The changes in gene expression can range from over expression of native proteins (e.g. Epidermal Growth Factor Receptor (EGFR), Survivin), mutated proteins (e.g. p53) to reactivation of oncofetal proteins (e.g. AFP, Glypican 3 (GPC3)) resulting in phenotypic as well as the genotypic changes in the cancer. In HCC, there are a number of genes that are reported to be commonly over expressed including: AFP expressed in 86% of HCC; GPC3 expressed in 83–90% of HCC (Kandil & Cooper, 2009) and Survivin expressed in up to 90% of HCC (Nassar et al., 2009).

The expression of a gene is controlled by a complex interplay of molecular factors that are active in a cell specific manner and show differential activity in response to environmental changes like tumor related hypoxia. Promoters are cis-acting elements generally present at the 5′-end of a gene of interest. Active promoters provide a binding site for transcription factors to bring about efficient gene expression (Forrest et al., 2014).

**Figure 3.**
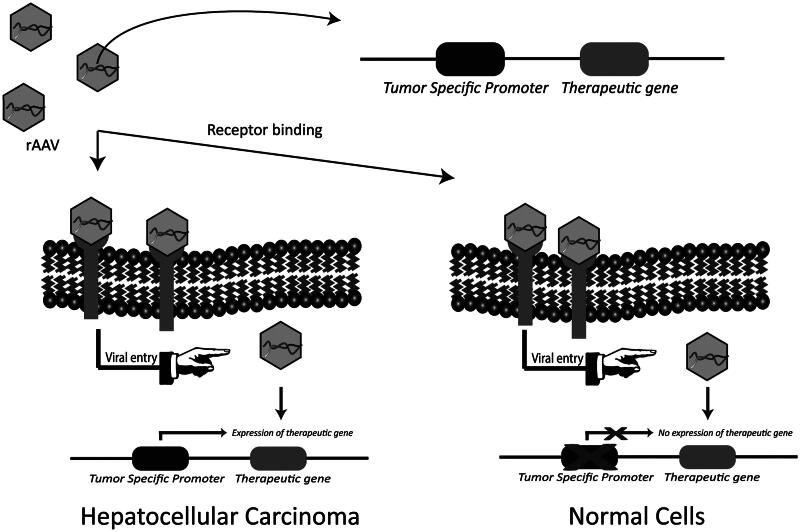
Transcriptional targeting with HCC-specific promoters. Tumor specific promoters are selectively active in cancer cells and are able to regulate the expression of therapeutic gene in a cancer specific manner. The identification and use of HCC-specific promoters could minimize off-target effects by limiting the expression of therapeutic gene in HCC.

In order to prevent expression of therapeutic transgenes in non-HCC cells and limit toxicity in normal cells, targeting at the level of transcription with these TSPs can be used (Figure 3; Robson & Hirst, 2003). The proof of efficacy of transcriptional targeting using TSPs has been documented for different cancer types including prostate cancer (Figueiredo et al., 2006; Coulter et al., 2010), breast cancer (Li et al., 2005), colorectal cancer (Li et al., 2009), non-small cell lung cancer (Pasini et al., 2015), melanoma (Lu et al., 2005) and ovarian cancer (Casado et al., 2001) using adenoviral vectors.

The first reported gene therapy for HCC using AAV vector was performed using an AFP promoter to drive the expression of HSV-TK to selectively kill HCC (Su et al., 1996). AFP is an oncofetal protein overexpressed in HCC and commonly used as a diagnostic marker (Forner et al., 2012). The use of AFP to selectively kill AFP-positive cells was later reproduced in human xenograft cancers in an athymic mouse model (Su et al., 1997). In similar studies, the human telomerase reverse transcriptase (hTERT) promoter, active in about 90% of cancer types including HCC (Kim et al., 1994; Iliopoulos et al., 2009), has been used for targeted cancer therapy (Abdul-Ghani et al., 2000; Gu & Fang, 2003). An AAV vector expressing pro-apoptotic factor TRAIL under the control of hTERT promoter was shown to be efficient in killing HCC both *in vitro* and *in vivo*. Moreover, minimal transgene expression was reported in normal cells including hepatocytes (Zhang et al., 2008; Wang et al., 2010). Survivin is another cancer specific promoter which has been used for suicide gene therapy of HCC cells (Qu et al., 2013). In another approach for transcriptional targeting, liver specific apoE alpha-1-antitrypsin (apoE-AAT) promoter was combined with cell-cycle-dependent elements (CDE) and cell-cycle genes homology regions (CHR) to achieve HCC-specific expression of luciferase (Sia et al., 2013). microRNA has also been used to transcriptionally target HCC. The binding region of HCC-specific miR122a was combined with a liver specific promoter in order to achieve HCC-specific HSV-TK expression mediated by AAV8 (Della Peruta et al., 2015). In another study, apoE-AAT was combined with sequences of miR122a for transcriptional targeting of HCC (Fu et al., 2012).

Given the heterogeneity of HCC, the identification of new or multiple transcriptional targets for gene therapy may be required. This may be achieved by screening and identification of active promoters, not functional in normal cells, through the use of publically available microarray data and/or though deep sequencing of patient biopsy samples (Foka et al., 2010). Finally, the combination of these two strategies, i.e. transductional and transcriptional targeting could act synergistically and enhance therapeutic outcomes compared to targeting with one approach alone. Reynolds et al. reported a 300 000-fold increase in the selectivity of transgene expression using adenovirus in lung after combinatorial vector targeting (Reynolds et al., 2001). The possibility and efficacy of combining these two strategies have been demonstrated with adenoviral vectors in colorectal cancer (Li et al., 2009) and ovarian cancer (Barker et al., 2003).

## Discussions

Developing effective therapies for HCC requires that we face both the biological and practical constraints of treating this disease. HCC develops in an environment of liver disease and is often characterized by cirrhosis and poor liver function. Treatments that are highly effective in killing the tumor may not be clinically appropriate in this setting, particularly if it leads to increased liver toxicity. Targeting treatments specifically to the HCC while sparing the normal/diseased liver may be required for an effective therapy for HCC. Gene therapy, which aims at correcting diseases at the genetic level, may be an alternative to conventional treatments. However, the success of gene therapy depends not only on developing a potent anti-cancer therapeutic strategy but potentially and maybe more importantly, on developing a vector to efficiently and safely deliver the therapeutic transgene.

AAV is arguably the best gene delivery vector at our disposal today. Numerous preclinical and clinical trials have shown the vector to have limited toxicity, minimal immune activation to the transgene and an ability to transduce a wide range of dividing and non-dividing cells to maintain long-term, stable transgene expression. However, these clinical trials have identified a few limitations of AAV vectors which need to be resolved for therapeutic benefits. Moreover, most of the AAV serotypes have a natural tropism for liver which presents a problem when targeting of HCC is required.

Fortunately, the current knowledge of AAV biology has identified key binding sites on the viral capsid and many of the cellular receptors which allow viral entry. Modification of AAV capsid without disruption of its ability to form an intact vector is one of the most promising areas of research currently under development. Using this strategy, AAV vectors with altered tropism and increased specificity for a number of different cell lines have been designed. These reports of transductional targeting of AAV suggest that similar techniques could be used for HCC in order to generate HCC targeted vectors. An additional layer of HCC specificity could be achieved by selecting tumor-specific promoters, specifically active in HCC, to drive the expression of the therapeutic transgene. This method of transcriptional targeting is highly likely to limit the off-target therapeutic gene expression.

The combination of both of these targeting strategies has the potential to overcome many of the limitations of AAV based gene delivery. First, the vector dose required to achieve therapeutic levels of transgene could be significantly lowered as most virus will transduce the tumor thereby limiting the potential for the development of capsid specific T-cell responses. Moreover, the capsid modified vectors have the potential to escape the preexisting NAbs and specifically transduce HCC while avoiding entry in the normal cells which could be of special interest in patients with liver cirrhosis. Finally, the off-target effects could be significantly minimized by using the TSPs in combination with capsid targeting. As more novel and reliable ligands and promoters, specific for HCC, are identified, the prospect of developing rationally designed targeted AAV vectors for HCC becomes closer to reality.
